# Can the impact of gender equality on health be measured? a cross-sectional study comparing measures based on register data with individual survey-based data

**DOI:** 10.1186/1471-2458-12-795

**Published:** 2012-09-17

**Authors:** Ann Sörlin, Ann Öhman, Nawi Ng, Lars Lindholm

**Affiliations:** 1 Department of Public Health and Clinical Medicine, Centre for Global Health Research, Umeå University, S-901 87, Umeå, Sweden; 2 Umeå Centre for Gender Studies, Umeå University, S-901 87, Umeå, Sweden

**Keywords:** Gender equality, Self-rated health, Companies

## Abstract

**Background:**

The aim of this study was to investigate potential associations between gender equality at work and self-rated health.

**Methods:**

2861 employees in 21 companies were invited to participate in a survey. The mean response rate was 49.2%. The questionnaire contained 65 questions, mainly on gender equality and health. Two logistic regression analyses were conducted to assess associations between (i) self-rated health and a register-based company gender equality index (OGGI), and (ii) self-rated health and self-rated gender equality at work.

**Results:**

Even though no association was found between the OGGI and health, women who rated their company as “completely equal” or “quite equal” had higher odds of reporting “good health” compared to women who perceived their company as “not equal” (OR = 2.8, 95% confidence interval = 1.4 – 5.5 and OR = 2.73, 95% CI = 1.6-4.6). Although not statistically significant, we observed the same trends in men. The results were adjusted for age, highest education level, income, full or part-time employment, and type of company based on the OGGI.

**Conclusions:**

No association was found between gender equality in companies**,** measured by register-based index (OGGI), and health. However, perceived gender equality at work positively affected women’s self-rated health but not men’s. Further investigations are necessary to determine whether the results are fully credible given the contemporary health patterns and positions in the labour market of women and men or whether the results are driven by selection patterns.

## Background

The unequal distribution of health in the population is of major interest for public health research and policy. The gender distribution pattern is well known: women live longer but report more ill-health than men [1]. This article examines whether gender equality in companies is associated with individuals’ self-rated health. Although examination of the relationship between gender equality and health is not new [2,3], to be able to answer this definitively both the concepts must be properly measured. Unfortunately, this is not easily achieved.

One of the reasons why it is difficult to conduct research on gender equality is that there is no generally agreed-upon definition of gender equality and no generally agreed way of measuring it [4]. In Sweden, although most people are positive towards increased gender equality, precisely what this means is not so obvious. People seldom define the concept in their daily talk. A common answer to the question “What is gender equality?” is “Equal pay for equal work” or “Sharing household duties equally”. And many Swedish people seem to consider that they are gender equal [5]. Despite its importance, a precise and generally agreed-upon definition of gender equality is difficult to identify: some researchers use the explicit term “gender equality” [6,7], while others use concepts such as fairness or equity [8,9]. The aim of this article, then, is to investigate and evaluate different concepts of gender equality in relation to self-rated health. In general, definition and measurement of equity can follow two different, but likely complementary, routes. The first is a more formal measurement of selected key aspects (e.g. income, position) from an external perspective. A typical research question in this tradition is whether differences in resources between the sexes per se affect health [10]. The second possible route is internal measurement: how do people perceive their situation? Do they perceive it as equitable or unequal? And does this perception affect their health?

That the availability of resources affects health is self-evident; this is revealed most clearly in a global perspective. However, beyond a certain level of material resources, the relationship may change. The direct material links between poverty and poor health are replaced by more indirect links between relative poverty and poor health. According to Marmot [11], people who perceive differences in resources and life opportunities as unfair may find their health affected. This idea has been explored mostly from a social class perspective, but it can also be relevant in a gender perspective. Women’s experiences of being treated unfairly in working life and other social situations may affect their health [12,13]. International comparisons show that high female employment in a country often also means high segregation between men and women, measured by a segregation index [14]. Segregation can be expressed not only as men and women working in different sectors, but also in terms of what they do at work **–** this can be seen both at sector level and within companies. The presence of gender**-**unequal companies is likely to have consequences in several spheres of life: profits, incomes, economic growth, health, working environments and peoples’ sense of fairness. Swedish men work in the private sector to a larger extent than women (81% to 48%), whilst Swedish women work to a larger extent in the public sector (50% versus 18%) [13]. The injustice that results from the division of labour between women and men affects virtually all women, men and children in society, though not all in the same way. Working life is significant in reinforcing the gender system [15,16]. In this article, we compare two measurements of gender equality and the possible relation of gender equality to self-rated health. The first measurement is the OGGI (Organizational Gender Gap Index), a register-based index measuring gender equality at organization level. We developed the index based on the Swedish national policy for gender equality [17].

The main objective of the policy is that women and men should have equal power to shape society and their own lives. A prerequisite is that women and men should have the same opportunities, rights and obligations in all spheres of life.

The interim objectives of the policy include:

1. Equal distribution of **power and influence**. Women and men should have the same rights and opportunities to be active citizens and to shape the conditions for decision making.

2. **Economic equality** between women and men. Women and men should have the same opportunities and conditions regarding education and paid work providing lifelong economic independence.

3. **Equal distribution of unpaid care and household work**. Women and men should take equal responsibility for household work and be able to give and receive care on equal terms.

4. Men’s **violence** against women should cease. Women and men, girls and boys, should have equal rights and opportunities to physical integrity.

This policy represents social objectives adopted by decision-makers, who occupy their positions as a result of a socially approved political process [18]. Thus, we believe that a definition based on official policy meets with “widespread approval”, at least at a normative level. We sought to conceptualize the keywords above in bold with variables found in public registers. All the variables occurred in official registers except one – men’s violence against women. There are of course statistics on violence in Swedish registers; however, there are no studies on the frequency of violence in the population as a whole. Further, the samples are too few in number to be able to be used in this study, where we employ individual data merged at organization level. Our aim of collecting data at company level would make it even more difficult to use the information on violence currently available. This lack of data was unexpected. Therefore, the keywords power and influence, economic equality, and equal distribution of unpaid care and household work were conceptualized using the following six variables: 1) numbers of men and women in the organization, 2) full-time vs part-time employment, 3) educational level, 4) income, 5) days on parental leave and 6) days on temporary parental leave. Although these variables do not provide a complete picture of gender equality in organizations, they do give a limited snapshot that can be compared across organizations and over time.

The production of statistics divided by sex has developed over time and a great deal is now available from various statistical providers. Interpretation, however, is not always straightforward [19], as the concept of gender equality is more extensive and difficult to grasp than a simple difference between men and women. Nevertheless, the statistical approach can provide a useful and necessary starting point, particularly when assessing policy implementations; it constitutes a useful base for gender equality analysis and development in organizations. The difference between the sexes can be presented as a gap – a common method in gender research [20,21]. This provides an easily understood representation of differences between the sexes, for instance in income or education.

The sense or perception of gender equality can also be measured by asking individuals about their views of gender equality. In our comparison of different measurements of gender equality, we used self-rated or self-perceived gender equality as our second measurement. Self-rated gender equality often measures the sense of fairness or views on traditional versus modern family constructs [22,23]. Self-rated gender equality at work is less frequently employed: we have not found any published paper describing the concept as used in our study.

Health is also a difficult concept to evaluate, with measurements of health perhaps even more diverse. The outcome measurement of self-rated health (SHR), commonly employed in this type of research, is also used in this article [24-26]. One of the most frequent methods of measuring self-rated health is a single question asking people to rate their overall health on a scale from excellent to poor. It is argued that this single (global) question provides a good summary of how people perceive their overall health [27,28]. Global self-rated health assessments are valuable because they are sensitive to health changes, capture broader dimensions of health than traditional diagnostic tools, and are easy to manage. The self-rated health indicator has been found to have good reliability [29-32] and is recommended by the World Health Organization [33].

In Sweden, 75% of the population consider their health to be “good” or “excellent”; only 5% consider their health to be “very poor”. Overall, self-rated health varies by age and sex, with men tending to declare higher rates for health than women across all age groups [13].

### Aim

The aim of this study was to investigate potential associations between two different measures of gender equality at work and self-rated health.

## Methods

### Study participants

The present paper forms part of a larger study on gender equality and health. The first part of the overall study was a register study, with the aim to construct an index for measuring gender equality in organizations [17]. The study population for the register study comprised two labour market sectors. The first, here called Sector 1, comprised 11 471 people in 46 companies. The second, here called Sector 2, included 32 151 individuals in 77 companies. The index was constructed on the basis of six variables and is called the Organizational Gender Gap Index or OGGI.

Means for companies, by sex, were calculated for each variable representing the three interim objectives of the gender equality policy. The ratio for each variable was calculated irrespective of whether it favoured one sex or the other, with the larger number always made the numerator. Some ratios were very high: one extreme example is parental leave, with a ratio of 42 to 1 in one company. To avoid giving one variable unreasonable weighting, we set 3 as the maximum, because this is twice the ratio limit of 1.5 (i.e. the 40/60 percentage rule) that we set for equality. Therefore, the sum for each variable could range from 1 to 3. The variables were added using equal weight and divided by 6. This gave a continuous scale from 1, full gender equality**,** to 3, least equality. Companies were counted as gender equal if they scored 1.5 or below.

In the next part of the study, we selected the most and least equal companies in the two sectors to participate in a survey. Selection by the index-ranking list gave a total of 21 companies. Six were ranked as gender equal and 15 as gender unequal**,** with altogether 2861 employees. The aim of the survey was to evaluate the differences in gender equality at company level in greater depth. All 2861 registered employees (1885 men and 976 women) in the 21 companies were invited to participate and complete a self-administered questionnaire. The response rate was 49.2%, giving a total of 1407 individual respondents, 875 men (62.2%) and 532 women (37.8%). The response rate differed between companies: in the six gender-equal companies from 29% to 83%, with a mean of 48.1%, and in the 15 gender-unequal companies from 8% to 92%, with a mean of 50.3%. The size of the companies ranged from 23 to 518 employees.

### Data collection

The questionnaire was constructed in collaboration with Statistics Sweden, who later administrated the data collection and database. The questionnaire was first tested for user-friendliness in a pilot study with 300 people from a separate cohort and subsequently revised with the help of experienced constructers at Statistics Sweden. The questionnaire included a total of 65 questions on education, income, employment status, share of household work, parental leave and health-related issues. A portion of the questions was addressed to both the respondent and his or her partner. The questionnaire was sent out by post, followed by two reminders.

Statistics Sweden was able to conduct a comprehensive analysis of non-responders, as the questionnaires were sent to individual employees of companies ranked by the register study, meaning a considerable amount of register data was available on those who chose not to participate. This analysis was presented to us in aggregated form. Non-respondents had different characteristics to respondents: they were more likely to be male (55% and 46% respectively of the selected women and men responded), young (the response rate among those aged ≤ 40 and over 40 was 39.5% and 52% respectively) and on a lower income (of those who responded, 51.3% earned at least SEK 310 000 per year, while only 41.6% of the non-responders earned the same amount).

### Gender equality indicators

The OGGI based on register data was used to rank the companies by gender equality; this was done as part of an earlier project and formed the basis for the selection of respondents in the present study. The index comprised six variables**:** numbers of men and women, educational level, income level, full-time vs part-time work, days on parental leave**,** and days on temporary parental leave. The purpose of the index is to measure the gap between men’s and women’s conditions in working life. “Gender gap” should be understood here as measured differences between the means for men and women aggregated at company level, in well-defined dimensions of gender (in)equality as set out in political goals. The index is based on Swedish public registers and investigates compliance with national policy. The index was dichotomized, dividing all employees into two groups: a) those working in a gender-equal company and b) those working in a gender-unequal company. The layout of the index varied depending on setting, but the current format was found to suit the Swedish context well.

Self-rated gender equality at work was assessed on a modified four-point categorical scale containing the response alternatives “completely gender equal”, “relatively gender equal”, “not very gender equal” and “not at all gender equal”. For the analysis we used three categories. The two response alternatives “not very gender equal” and “not at all gender equal” were merged into a single category because of the small numbers in each category. The outcome measure of self-rated health was dichotomized into “good” and “poor” health.

### Data analyses and statistical approach

As individuals working at the same company might not be independent of each other in the variables used in this analysis, we used two-level multi logistic regression analyses, in which individuals (level 1) were nested within each company (level 2). Two multilevel logistic regression analyses were conducted separately to assess the associations between (i) self-rated health and the register-based company gender equality index, OGGI, and (ii) self-rated health and self-rated gender equality at work**.** The analyses were adjusted for age, education, income, and full-time vs part-time employment. The second analysis using self-reported gender equality at work was also adjusted for the OGGI. We estimated the variance at the 2^nd^ level and the variance participation coefficient, which indicates the proportion of variance in the outcome variables explained by the higher level (companies). All the data analyses were conducted using STATA Version 12 (StataCorp 2012).

### Ethical considerations

Ethical approval was obtained from the Regional Ethics Review Board in Umeå (D-nr 06-156 M).

## Results

Different results were found for the OGGI and gender equality as perceived by employees. There was no association between the OGGI and self-rated health; however, for the perceived gender equality measurement there was an association between women’s self-rated health and gender equality. Table 1 presents a number of descriptive background variables.

**Table 1:** Distribution of study subjects

**Variables**	**Men**		**Women**	
	**Working in gender-equal companies (n = 303)**	**Working in gender-unequal companies (n = 572)**	**Working in gender-equal companies (n = 316)**	**Working in gender-unequal companies (n = 216)**
Age group (%)				
<30	27 (8.9)	102 (17.8)	27 (8.5)	51 (23.6)
31-50	212 (70.0)	328 (57.4)	201 (63.6)	120 (55.6)
>50	64 (21.1)	142 (24.8)	88 (27.9)	45 (20.8)
*X*^2^ (p-value)	16.96 (p < 0.001)		23.77 (p < 0.001)	
Education (%)				
Secondary	31 (10.4)	77 (13.7)	68 (21.9)	27 (12.6)
Further	117 (39.4)	229 (40.6)	132 (42.4)	87 (40.7)
Higher	149 (50.2)	258 (45.7)	111 (35.7)	100 (46.7)
*X*^2^ (p-value)	2.48 (p = 0.290)		9.93 (p = 0.007)	
Income, monthly (%)				
< 20,000	18 (6.0)	54 (9.6)	114 (36.3)	57 (26.8)
20,000 – 30,000	89 (29.7)	222 (39.4)	107 (34.1)	82 (38.5)
> = 30,000	193 (64.3)	287 (51.0)	93 (29.6)	74 (34.7)
*X*^2^ (p-value)	14.48 (p = 0.001)		5.31 (p = 0.07)	
Employment (%)				
Full time	291 (96.7)	516 (91.5)	239 (76.8)	149 (70.3)
Part time	10 (3.3)	48 (8.5)	72 (23.2)	63 (29.7)
*X*^2^ (p-value)	8.45 (p = 0.004)		2.84 (p = 0.092)	
Self-rated equality at company				
Completely equal	103 (34.4)	215 (38.5)	50 (16.0)	57 (27.3)
Quite equal	161 (53.9)	259 (46.4)	191 (61.2)	120 (57.4)
Not equal	35 (11.7)	84 (15.1)	71 (22.8)	32 (15.3)
*X*^2^ (p-value)	4.64 (p = 0.098)		11.52 (p = 0.003)	

The higher proportion of young men and women in gender-unequal companies (p < 0.001) is notable. Women in gender-unequal companies had a higher level of education than women in gender-equal companies (p < 0.001). A greater proportion of men in gender-equal companies had a higher income compared to men in gender-unequal companies. In contrast, though not statistically significant, there was a larger proportion of women in gender-unequal companies on higher incomes. Finally, there were differences in working time, with a significantly higher proportion of men working part time in gender-unequal companies. In the last section of the table, the two measurements of equality are compared; they do not coincide – compared to women working in gender-equal companies a significantly higher proportion of women working in gender-unequal companies rated their company as totally equal (27.7% vs 16% p ≤ 0.001).

### Association with health

The association between gender equality at work and self-rated health was assessed using two methods of measuring gender equality, namely the OGGI (Table 2) and self-rated equality at the company (Table 3). Table 2 shows no statistically significant differences in how men and women in either gender-equal or gender-unequal companies reported their health. About 80% of men and 75% of women in gender-equal companies reported their health as “good”; the corresponding figures for gender-unequal companies were 82% and 79% respectively. Though not statistically significant, the multivariable logistic regression revealed that both men and women in gender-equal companies had lower odds of reporting good health than those in gender-unequal companies, even after adjusting for other sociodemographic variables. The VPC indicated that 3.8% and 7.5% of variances in self-rated health in men and women respectively were explained by the company level. The variance at company level decreased when individual sociodemographic variables were added to the regression analysis, indicating that self-rated health did not differ among individuals at the same company.

**Table 2:** Association between gender equality index and self-rated health in a multilevel logistic regression analysis

**Variables**	**Self-Rated Health**			
	**Men**		**Women**	
	**Good health**	**Poor health**	**Good health**	**Poor health**
**Type of company, n(%)**				
Working in a gender-equal **company**	239 (79.7)	61 (20.3)	234 (74.8)	79 (25.2)
Working in a gender-unequal **company**	466 (82.2)	101 (17.8)	170 (78.7)	46 (21.3)
**Null model**				
Variance estimate (VPC)	0.1295 (3.8%)		0.2660 (7.5%)	
**Univariate model**				
Unadjusted OR (95% CI)				
Working in a gender-equal **company**	0.81 (0.52-1.26)		0.84 (0.50-1.43)	
Working in a gender-unequal **company**	1		1	
Variance estimate (VPC)	0.1589 (4.6%)		0.2475 (7.0%)	
**Multivariable model**				
Adjusted OR (95% CI) *				
Working in a gender-equal **company**	0.82 (0.56-1.19)		0.99 (0.63-1.55)	
Working in a gender-unequal **company**	1		1	
Variance estimate (VPC)	1.97x10-7 (0%)		1.81x10-8 (0%)	

Note: *OR* Odds Ratio, *VPC* Variance Partition Coefficient, estimated by variance/(variance + 3.29). * Adjusted for respondent’s age, education level, income and employment level.

**Table 3:** Association between self-rated equality at company and self-rated health in a multilevel logistic regression analysis

**Variables**	**Self-Rated Health**			
	**Men**		**Women**	
	**Good health**	**Poor health**	**Good health**	**Poor health**
**Self-rated equality at company, n(%)**				
Totally equal	268 (84.5)	49 (15.5)	87 (81.3)	20 (18.7)
Quite equal	340 (81.1)	79 (18.9)	247 (79.9)	62 (20.1)
Not equal	86 (72.3)	33 (27.7)	62 (60.2)	41 (39.8)
**Null model**				
Variance estimate (VPC)	0.1295 (3.8%)		0.2660 (7.5%)	
**Univariate model**				
Unadjusted OR (95% CI)				
Totally equal	2.10 (1.27-3.47)		2.80 (1.47-5.36)	
Quite equal	1.65 (1.03-2.64)		2.60 (1.59-4.25)	
Not equal	1		1	
Variance estimate (VPC)	1.73x10-8 (0%)		0.1167 (3.4%)	
**Multivariable model**				
Adjusted OR (95% CI) *				
Totally equal	1.65 (0.96-2.85)		2.58 (1.34-4.96)	
Quite equal	1.52 (0.91-2.52)		2.62 (1.58-4.36)	
Not equal	1		1	
Variance estimate (VPC)	9.88x10-7 (0%)		4.98x10-8 (0%)	
**Multivariable model**				
Adjusted OR (95% CI) **				
Totally equal	1.65 (0.96-2.85)		2.64 (1.36-5.13)	
Quite equal	1.54 (0.92-2.57)		2.64 (1.59-4.40)	
Not equal	1		1	
Variance estimate (VPC)	8.03x10-8 (0%)		6.69x10-9 (0%)	

Note: *OR* Odds Ratio, VPC Variance Partition Coefficient, estimated by variance/(variance + 3.29). * Adjusted for respondent’s age, education level, income and employment level. ** Adjusted for respondent’s age, education level, income, employment level and type of company based on the gender equality index.

Table 3 shows that, compared to women who perceived their company as “not equal”, women who perceived their company as “totally equal” or “quite equal” had higher odds of reporting “good health**”** (OR = 2.64, 95% confidence interval = 1.36 – 5.13 and OR = 2.64, 95% CI = 1.59-4.40). Though not statistically significant, we observed the same trends in men after adjusting for age, highest education level, income, employment level and type of company based on the gender equality index.

## Discussion

With the register-based index, OGGI, we could not find any association between equality in companies and self-rated health. However*,* the odds for good health were 2.8 times higher for women who rated their company as gender equal; we could not find any statistically significant association for men. Why, then, is perceived gender equality in the workplace associated with good self-rated health for women but not for men?

We would like to put forward two possible explanations. As already stated**,** men have a shorter life expectancy than women**,** and according to the convergence hypothesis [5] more gender**-**equal conditions in both the private and public spheres would decrease male mortality but increase male morbidity [34]. The theory of convergence should in this case mean that the differences in health outcomes, mortality and morbidity, which today show different patterns for men and women, would converge with increased gender equality – i.e. men would live longer and women would feel better. The gender differences we see today have various explanations, sometimes biological, sometimes more lifestyle related. Men’s shorter life expectancy is often explained by risk-taking, such as excess alcohol consumption, smoking, driving at speed and so on [35]. It is well established that men seek help later than women and that this negatively affects their health. The social constructions of traditional masculinity are usually considered a disadvantage to men’s health [36]. Further, it has been argued that women are taught to be more aware of their bodily functions and therefore more likely to discover ill-health and hence seek help more often and sooner than men [37]. Women’s greater ill-health and higher levels of sickness absence are hence explained in many ways: some explain it as a way of handling the dual burdens of paid and unpaid work; others view it as constituted by the gendered structures of society; and yet other explanations cite the biological differences between men and women. The adjusted convergence theory implies that when gender equality increases and men and women have greater equality in their possibilities to shape their lives and society, more gender-equal health will follow – men will live longer and women will live healthier lives [38]**.**

Further, men are considered to benefit more from an unequal situation at work, which might explain why they do not perceive differences between men’s and women’s conditions as a threat to health. If men lose privileges in their working life, it does not necessarily affect self-reported health positively.

Individuals’ experiences of gender (in)equality are most likely linked to health [39]. To be overlooked, or discriminated against, in career and/or salary based on sex most likely increases the risk for poor health. Marmot [40] argues that socioeconomic position is an important determinant of health. His results hold even if controlled for the effects of income, education and lifestyle risk factors such as smoking. Marmot’s causal relations are explained by the benefits of “being in control” of one’s own life. This could be part of the explanation for the differences we see in this study between men and women.

The external assessment and individuals’ own perceptions of gender equality or inequality seem not to coincide. One possible explanation could be that in large organizations employees are not likely to be aware of the overall structural conditions but rather base their assessment on “micro” experiences, i.e. the gender climate in their immediate working environment. One could reasonably argue that employees are familiar with their own working conditions and those of their closest colleagues, but less so for the company as a whole. Companies that deviate positively from the general gender pattern should perhaps develop an internal information strategy, as this kind of data is not easy accessible to individual employees. Being aware of gender equality at work may even positively influence the micro climates in the company. The index can thus be used to reveal facts that employees are not aware of. This is also a reason for conducting a more formal analysis from an external perspective – it can reveal patterns not easily detected from the individual perspective.

Another important finding of our study is the discrepancy between the OGGI and self-rated equality in companies. We found a significantly larger proportion of women in gender-unequal companies who rated their company as equal. Though not significant, almost 40% of men in gender-unequal companies gave the same rating. An explanation might be that, in judging equality at their company, respondents in our study used other criteria than the indicators used for OGGI. The index is based on an interpretation of official policy, and it is known that the practices are paternalistic in one respect – how parents do share parental leave and temporary parental leave. All the major political parties in Sweden agree on the goal that parents should share parental leave equally. However, there are very considerable differences of opinions on how to achieve this: some parties campaign for a couple’s unrestricted right to decide the division themselves, thus hoping for voluntary change [41], while others argue for more or less far-reaching legislation [42]. However, even those parties that favour legislation are somewhat reticent, as this issue is controversial even among their traditional supporters. Thus, companies with this traditional pattern of parental leave are given a relatively low rank based on the register-based index interpreting policy implementation, while employees working in those companies might be very happy with the situation, interpreting it as a sign of gender equality*.*

This index is constructed from official registers in Sweden**,** a country well known for its collection of data, where registers are complete, extensive and accessible for research. Access to the same kind of linked data might be limited in other settings. Research using register data will**,** we believe**,** be better accepted if it is recognized as a first step in an analytical process aiming to better understand gendered structures in organizations.

However**,** the limitations of OGGI are balanced by its strengths. Not only is comparison of different organizations easy and affordable using this method, it also provides an opportunity to follow developments year by year in individual organizations.

One limitation of this study might be the selection of companies. We set out to compare those companies that were ranked as gender equal with those ranked as gender unequal using our index. We decided to invite approximately 3000 persons in total, 1500 from gender-equal companies and 1500 from gender-unequal companies. However, as few companies were ranked as gender equal on our index, we were unable to invite as many respondents from gender-equal companies. Additionally, fewer women were invited to participate, as fewer women work in the private sector. Nevertheless, the response rate for women was higher, which is normally the case in Sweden. We also had large differences in response rates between companies, from 8% to 92%. The differences were somewhat less in gender-equal companies, from 29% to 83%. We have no good explanation for the large differences. The company with the lowest response rate, 8%, had 23 employees, 21 men and two women. The sole respondent was a woman. The company with the highest response rate, 92%, employed 47 men and 11 women. At this company, 53 people answered the questionnaire. There was also one company, with a response rate of 11%, that had grown from 28 to 120 employees in four years, which might have influenced the rate of response. Our overall reflection is that we do not know in what way the large differences in response rate influenced the outcome. As the non-responders tended to be male, young and on lower incomes, those who responded might perceive their company as more equal, and might have better attitudes towards their working life and health. Hence, the discrepancy between the index and self-rated equality might be explained by these factors.

One of the aims of this study was to investigate whether a single question could provide an answer to the question of how gender equal a couple’s relationship is. The question evaluated was **“**How gender equal is your workplace?” One single question that is often used is self-rated health, which has been shown to be a good predictor of mortality and morbidity. We thus wanted to know whether it was possible to evaluate gender equality in the same effective way. If this single question on gender equality gave the same answer as a battery of variables, then the single question could be used. Our subsequent question was whether self**-**perceived gender equality gives a reliable answer concerning levels of gender equality? We do not think it does. It is not likely that individuals can appraise the entire picture when the question concerns equality at organisational level. Each person will reflect those aspects that most concern them and probably from their own perspective. On the other hand, however, no index can ever describe an individual’s situation.

Women who perceived their workplace to be quite or totally equal had higher odds of reporting good health than women who did not perceive their workplace to be equal. The results of this study therefore suggest that gender equality is beneficial for women’s health. And gender equality does not seem to be bad for men. The construction of masculinity, as described above [35,43], may influence sickness absence patterns. In an environment where men, like women, are expected to participate in the upbringing of young children and stay at home when children are sick, it might also be more acceptable for a man to stay at home when he himself is sick (personal communication, Connell 2010). Although the changes in mortality patterns are obviously connected to shifts in smoking and drinking patterns, one could argue that steps towards increased gender equality are an underlying cause. Being a present and committed father might mean reducing mortality risks [44]. This development can already be seen in men participating in their children’s upbringing [45], and the long-standing life expectancy gap between men and women appears to be closing in many societies. Men take better care of their health than before and lead less risky lives, while women adopt more traditionally masculine behaviours such as smoking and alcohol consumption [46].

To summarize, then, measuring gender equality is an important but contentious and challenging task. Nevertheless, it is necessary for policymakers, politicians, researchers and many others to monitor gender equality, and valid measures need to be developed to enable comparison and use in future policy making. In order to measure gender equality correctly, instruments must be designed, tested and validated. Our attempt in this study to compare different types of gender equality measures in relation to health is one contribution to this development. The results of our study are credible considering the contemporary health patterns and positions of the sexes in the labour market.

The OGGI can be used to monitor the development of gender equality in Swedish society and its possible impact on health. Longitudinal research at organizational level could contribute to the development of the field.

## Conclusions

The dissimilarities between the measures for gender equality need to be further elaborated. Differences are not in themselves proof that one measurement is better than another. Disregarding information on normative levels and using statements from individuals only will mean we lose important data on the practical outcome of daily decisions. People do their best to make life work**,** both at home and at work. This will inevitably involve taking decisions that feel appropriate today**,** but that do not contribute to “equal power to shape society and one’s own life” in the long run**.** The normative indices are there to make us see what we are too close to notice. Register data can contribute to a broader picture; however, it is essential to use more than one method in the research of gender equality.

## Competing interests

The authors declare that they have no competing interests.

## Authors’ contribution

AS is the corresponding author and is also responsible for the database and calculations. AS, AÖ, NW and LL outlined and wrote the article together. All authors read and approved the final manuscript.

## Pre-publication history

The pre-publication history for this paper can be accessed here:

http://www.biomedcentral.com/1471-2458/12/795/prepub
